# Lectures on Military Surgery

**Published:** 1861-11

**Authors:** E. Andrews

**Affiliations:** Professor of Surgery, Surgeon to the Mercy Hospital; to the Chicago Dispensary, etc.


### CHICAGO MEDICAL EXAMINER

N. S. DAVIS, M. D., Editor.---FRANK W. REILLY, M. D., Ass't Editor.

VOL. II.] NOVEMBER, 1861. [NO. 11.

### Original Contributions.

ARTICLE I.

### LECTURES ON MILITARY SURGERY:

DELIVERED DURING THE SUMMER COURSE OF THE MEDICAL DEPARTMENT OF LIND UNIVERSITY.

By E. ANDREWS, A. M., M. D.,

PROFESSOR OF SURGERY; SURGEON TO THE MERCY HOSPITAL; TO THE CHICAGO DISPENSARY, ETC.

### LECTURE SEVENTH.

### SCURVY.

When men are confined for a long period to a diet, exclusively salt, especially if it is accompanied with a total absence of fresh vegetables, they are apt to become scorbutic. Hence, camp life occasionally gives rise to this disease, and it requires the full consideration of the surgeon. It is disputed whether scurvy is caused by excess of salt, by deficiency of fresh vegetables, or by deficiency of potash in the diet. Each of these theories has its advocates, but the limits of the present lecture, do not permit a full discussion of their merits. Fortunately, there is no obscurity in the practical directions for preventing and curing this disease, and indeed it is now almost banished from the public service.

Formerly, this pest scourged the crews of almost all vessels on long voyages, and the camp of almost all armies. So disastrous was it, that a man-of-war would often have scarcely sailors enough on duty to work the vessel into port. In voyages to the polar regions it was particularly troublesome, and the prevalence of cold, darkness and hunger were there considered causes of its onset. Dr. Hays, the surgeon of Dr. Kane's expedition, made some interesting observations in the polar regions upon the causes of scurvy, which are worthy of remark. In the crew of the *Advance*, it was observed that scurvy afflicted them whenever they were confined to a salt diet, but not otherwise. On one occasion when a party was sent out for a long trip in the darkness, the following facts were noted. The men, thus sent out, ran out of provisions and were obliged to subsist on fresh, raw walrus-meat, and only a starvation supply of that. They slept in the open air, and were exposed not only to cold and darkness, but to exhausting labor and starvation, yet no scurvy appeared among them. They also ascertained that the Esquimaux, who are often reduced to famine, but who subsist entirely on fresh food, never have scurvy. During all this time, those who remained in the vessel, free from hard labor, well clad and warm, and abundantly supplied with salt food, suffered severely from the disease. Among public institutions, such as prisons, orphan asylums, etc., the disease sometimes appears, even when no remarkable amount of salt is consumed, if the supply of potatoes or other fresh vegetables is neglected.

In practical management, the following conclusions are established: First—if men are kept solely on salt food and have no fresh vegetables, scurvy will always appear, after a time. Second—any depressing influences, such as cold, hunger, over-exertion, darkness, debility, or despondency will predispose to the attack, but if the corps has a full supply of fresh meat, bread and vegetables, then scurvy will not be troublesome.

This very statement shows what is to be done. Among the vegetable productions, lemons and potatoes have the highest anti-scorbutic reputation. Lemon juice is taken to sea in large quantities, and successfully given to the men in the navy, as a prophylactic. In the army, potatoes have been lately added to the rations, which is undoubtedly a highly important improvement. In our western territories the officers of our army have found the wild artichoke to be an effectual prophylactic and remedy for scurvy, and they also report that the Indians who live, in winter, on buffalo meat, and without salt, and on dried berries, never have the disease.

*Symptoms.*—Scurvy is manifested by a pale countenance, depression of spirits, a great muscular debility, and unusual indisposition to exertion. One early symptom, is a spongy, swollen condition of the gums, with a tendency to bleed upon the slightest touch. In bad cases, teeth become loosened, and even drop out. The tissues of the whole body seem to be in a state of starvation, so that absorption predominates over nutrition. Hence the walls of the blood-vessels become thinned and weakened, so that spontaneous extravasations of blood occur to a great extent, which coagulate and form hard lumps in the flesh. The same poverty of nutrition appears in other organs. Thus old cicatrices, which are imperfectly supplied with blood are entirely absorbed, leaving the wound to open again. Pains occur in various parts of the body, hemorrhages from the mucous canals are common, and fainting takes place on the slightest exertion. Various complications may intervene, and ultimately death occurs from exhaustion or from syncope.

*The treatment* of this disease is mainly dietetic. The patient must be taken at once from his salt rations and kept on fresh food of all kinds, and especially fresh vegetables and fruits. In this way recovery is almost certain. Special complications may be treated on general principles as they arise, and tonics may be given to increase the strength. On the whole, there is no branch of medicine which yields more certain results than the established treatment of scurvy.

### DIARRHŒA.

In all armies in active service, diseases of the bowels are the principal causes of admission to the hospitals.

Diarrhœa and dysentery slay five-fold more than the sword. The causes of this singular fact seem to be as follows: The men in volunteer armies are brought to a complete change of diet, which always tends to excite activity in the mucous coat of the intestines. To this is generally added a change of climate, often from cold to hot, which acts precisely in the same manner as a change of food. The necessity also of sleeping upon the damp ground, the constant exertion of marching and drilling in the hot sun, and the fact that nearly all campaigns are in the summer season, are abundant reasons for the fact that almost every recruit, within two months of entering service, has an attack of diarrhœa of greater or less severity. Miasmatic influences, and imprudent indulgence in eating and drinking, also come in as occasional causes.

The treatment of camp diarrhœa is different with different surgeons. Dr. Tripler, Medical Director of the Department of the Potomac, and an old army surgeon, is partial to using saline cathartics. When the patient is first seen, he simply gives from half an ounce to an ounce of sulphate of magnesia. After this has operated, the bowels remain quiet for a time, and in mild cases no further interference is necessary. If, however, the case is more severe, he gives opiates and astringents after the operation of the medicine, to prolong as much as possible the stage of quiet. When the tendency to discharges begins to return, he administers another dose of the cathartic, followed, if requisite, by opiates and astringents again. In many cases, he gets along with nothing but one, two, or three doses of the magnesia, no opiates being used. In general, it will be found that the newer the recruit, and the freer the climate from malaria, the freer is the case from typhoid tendencies, and the simpler the treatment required; while the longer the troops are crowded together in unhealthy camps, and the more malarious the climate, the more the disease takes on an epidemic character, and the more unmanageable and terrific does it become in its ravages. Mercurials must be used with discretion, and with proper regard to circumstances. In vigorous new recruits, especially in malarious western regions, a little blue mass often assists very much in the cure.

The following prescription does excellently for such cases:

| | | |
|---|---|---|
| ℞ | Mass. Hydrarg, - - - | grs. xv. |
| | Acet. Plumb, - - - | grs. xx. |
| | Pulv. Opii, - - - - | grs. xv. |

M. ft. pil. No. xx. Take two after every discharge.

In the really epidemic bowel diseases which break out frequently in camp, mercurials must be used sparingly, as the cases are of a typhoid character, and the depressing influence of these articles is to be avoided. In short, the surgeon must study the type of the disease, and the diathesis of the men, and adapt his treatment to the particular condition existing at the time. In general, if a diarrhœa is at all obstinate, it will be found impossible to restrain it permanently by opiates and astringents alone. The secretions of the mucous membrane are depraved and irritating in their character, and when they accumulate they increase the inflammation of the alimentary canal. Whenever this occurs, the bowels, though previously made quiet by the treatment, begin again to act. The surgeon, without waiting for the irritation to return to its full height, should clear out the *primæ viæ* by a gentle cathartic, such as sulphate of magnesia, castor oil or rhubarb. The operations of these medicines are by no means as exhausting as the discharges produced by the disease, and after they are over, the bowels are again favorably disposed for a season of quietude, which may be prolonged by opiates and astringents. In this way, alternately moving the bowels by medicine whenever the irritation demands it, and quieting them by suitable means in the interval, the case will generally progress to a rapid cure. During treatment, the patient should remain at rest, either in hospital or in quarters, and not be allowed to go out of camp nor be put on duty.

In the chronic stage, or in adynamic cases, the turpentine emulsion will be found very useful. A combination of turpentine emulsion, strychnia and nitric acid is highly efficient. The following is a convenient formula:

| | | |
|---|---|---|
| ℞ | Ol. Terebinth., - - - | ℥i. |
| | Acid. Nit. dilut., - - | ℥ss. |
| | Strychniæ, - - - - | grs.ij. |
| | Syrupi, - - - - | ℥jss. |
| | Gum. Acac., - - - | ʒij. |

M. fiat emulsio. Dose: a teaspoonful every three hours.

The turpentine and other stimulating remedies of course must not be given in the stage of active irritation, but only in the diarrhœa of debility.

DYSENTERY.

This disease consists, essentially, of an inflammation of the coats of the colon and rectum. The same causes that produce diarrhœa may also result in dysentery, and accordingly we find that it is one of the most fearful scourges of camp life. It is marked by frequent discharges from the lower bowel, accompanied with griping pains, and tenesmus, bringing away abundance of transparent mucous stained with blood. The severity of the suffering is often very great. In bad cases, the disease assumes all the constitutional characters of typhoid fever. The tongue becomes dry and brown; a low fever, with delirium may supervene, accompanied by feeble pulse and disturbed respiration. In the more adynamic cases, the tenesmus at length ceases, and the tenacious mucous is no longer secreted, but a mixture of blood and foul serous fluid escapes from the anus without straining, or even consciousness on the part of the patient. In some cases the disease is epidemic, typhoid in type, and terribly fatal. If it runs on to the chronic stage, there is generally ulceration of the bowels, and almost always a fatal result.

The treatment in mild cases is almost identical with that of diarrhœa. We find it necessary, however, to use opium freely to subdue the pain, and to prevent the excessive and irritating activity of the muscular coat of the bowel. The same principles as to the use of alteratives, astringents, cathartics, terebinthinates and tonics, will guide you here as in diarrhœa. There is, however, one resource which is peculiar to dysentery, and that is the use of injections. The inflamed surface of the colon is actually as accessible to topical applications as though it were on the external surface of the body. In the more sthenic cases, however, the muscular activity of the bowel is so great that injections are not well borne. The distention excites violent contractions and pain, and thus does more harm than good; but in the adynamic cases marked by feeble contractions, and a disposition to discharge blood and serum without much effort or pain, injections are well borne and very efficient. In such cases, large clysters of solution of nitrate of silver, forty grains to the ounce, have been thrown up not only without injury, but with decided benefit. This has, however, seemed to me a rather harsh treatment, and I have used, in preference, strong solutions of alum and tannin with the most gratifying effect. A single injection, after the following formula, sometimes stops the discharges of blood permanently:

℞ Acid. Tannic., - - - grs. xx.
Aqua, - - - - - Oj.
Aluminis, q. s. ut solutio saturata sit.

This is to be injected in large quantity, and if requisite, retained in the bowel for a few minutes, by pressing a napkin firmly against the anus.

As in diarrhœa, the utmost quiet must be enforced upon the patient, and the peculiarities of type and diathesis thoroughly considered in shaping the treatment.

THE OTHER medical diseases which occur in camp life, do not require a separate consideration, as their treatment is the same as in private practice. The only point about them to which I would direct your attention is the fact, that they are very often epidemic, and have the typhoid character common to most other epidemics.

---

I HAVE now completed my course of lectures upon Military Surgery. In such a brief series, I have necessarily omitted many things which it would be of great interest to discuss; nevertheless I have set before you all the main points of military practice. I have displayed to you, upon the dead subject, all the remarkable peculiarities of gun-shot wounds, and given a brief but tolerably comprehensive outline of the chief accidents common in war, and of the principal diseases prevalent in camps. I trust that the attention you have given these topics, will fit you better for the service, and that in some coming hour, on the battle field, you will realize practical advantages from the discussions we have held upon these important themes.

